# Enhanced Steam Temperature Enabled by a Simple Three‐Tier Solar Evaporation Device

**DOI:** 10.1002/gch2.202000092

**Published:** 2021-02-18

**Authors:** Zhenzhen Guo, You Xu, Fang Yu, Jiacheng Yin, Xianbao Wang

**Affiliations:** ^1^ Key Laboratory for the Green Preparation and Application of Functional Materials Ministry of Education Hubei Key Laboratory of Polymer Materials School of Materials Science and Engineering Hubei University Wuhan 430062 China

**Keywords:** high‐temperature steam, high‐quality water, solar evaporation, three‐tier devices

## Abstract

Interfacial water evaporation technology by using solar energy provides one of the promising pathways for freshwater shortage management. However, current research mainly focuses on the improvement of evaporation efficiency by macro or microregulations, ignoring the steam temperature, which is a manifestation of the quality of water. Herein not only is a high‐rate solar evaporation achieved but also steam temperature is enhanced by a simple three‐tier (wet absorber–air gap–dry absorber) device. In a routine interfacial evaporation test, the evaporator achieves a stable evaporation rate up to 2.15 kg m^−2^ h^−1^ under one sun, demonstrating a competitive evaporation rate compared with other reports. With the three‐tier device, the steam temperature can increase 33.7%, 41.13%, and 47% without dry absorber under one sun, two sun, and three sun illumination, respectively. At the same time, the steam temperature can be as high as 95.5 °C under three sun intensities. This work provides the possibility of using a simple three‐tier device for high‐temperature steam generation without extra energy input, which contributes to an idea for future research on the production high‐quality water.

## Graphical abstract

MoS_2_@Cu_9_S_5_ is developed as a photothermal material for solar evaporation. The evaporation rate can reach up to 2.15 kg m^−2^ h^−1^ and the water produced can reach up to 16.5 kg m^−2^. Further, a simple three‐tier device is designed for enhanced steam temperature. The steam temperature can increase 33.7%, 41.13%, and 47% under one sun, two sun, and three sun illumination, respectively.

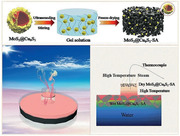

## 1 Introduction

Most of the world's water cannot be used directly as drinking water due to various water pollution and high concentration of salt in seawater, so the shortage of clean water is an urgent problem to be solved.^[^ ^1^, ^2^ ^]^ Recent years, the interfacial water evaporation technology (IWET) relying on photothermal materials and solar energy has gradually stepped into public views for solving the problem of clean water shortage.^[^ ^3^, ^4^, ^5^, ^6^, ^7^, ^8^ ^]^ With regards to the studies on high‐efficiency evaporation and sewage treatment, researches have made good progress through macrostructure control, including thermal management (weakening of heat radiation and conduction,^[^ ^9^, ^10^, ^11^, ^12^, ^13^ ^]^ environmental heat utilization^[^ ^14^, ^15^, ^16^ ^]^), water channel designs (1D water channel,^[^ ^17^, ^18^, ^19^ ^]^ 2D water channel,^[^ ^20^, ^21^, ^22^ ^]^ hydrophilic porous aerogel or hydrogel,^[^ ^23^, ^24^, ^25^, ^26^ ^]^ etc.), and hydrophile‐hydrophobic control designs.^[^ ^27^, ^28^, ^29^, ^30^ ^]^ However, the researches on high‐temperature steam at low solar concentrations involves less and remains a highly challenging issue. High‐temperature steam not only can improve the quality level of the collected water, but it can expand its own applications, such as sterilization and disinfection. Therefore, the realization of high‐temperature steam generation has important development prospects in future researches.

Since the direct contact between water and the photothermal materials causes a cooling effect, resulting in a low surface temperature, the generation of high‐temperature steam by a simple IWET under low solar intensities is difficult to achieve. Therefore, in order to achieve high‐temperature steam, it usually requires light concentrators to increase the intensity of the sun by ten times. In recent years, researchers have also made some efforts for high‐temperature steam generation. Especially, Chen's group made good progress in the study of high‐temperature steam generation. For example, in 2016, Chen's group achieved 100 °C solar steam generation under one sun by thermal concentration.^[^ ^31^ ^]^ In 2018, they developed a contactless system for superheating steam generation under one sun.^[^ ^32^ ^]^ But the evaporation efficiency of these device is low due to the low evaporation area or noncontact heating, and the preparation process of these systems is complicated. Therefore, in the interfacial solar evaporation system, how to use simple systems to realize the solar steam generation with enhanced steam temperature is still worthy of study, which will also increase some ideas for the subsequent researches to deeply study ultrahigh‐temperature steam by a simple method in the future.

In this work, we not only achieve the high‐rate evaporation, but based on the IWET, we design a simple three‐tier (wet absorber–air gap–dry absorber) evaporation device for solar steam generation with enhanced steam temperature. Here, we choose MoS_2_@Cu_9_S_5_ composite as the photothermal material, which shows perfect light absorption performance in the entire solar spectrum range. With sodium alginate (SA) hydrogel as the 3D framework, in the conventional photothermal interfacial evaporation test, the photothermal evaporation rate of MoS_2_@Cu_9_S_5_‐SA can be as high as 2.15 kg m^−2^ h^−1^ under one sun, demonstrating its excellent photothermal performance. On this basis, we compare the steam temperatures with the three‐tier evaporation device and without a dry absorber. With the three‐tier device, the steam temperature can improve 33.7%, 41.13%, and 47% of the device without dry absorber under one sun, two sun, and three sun illumination, respectively. Meanwhile, the evaporation rate with the three‐tier device can be kept at 1.04 kg m^−2^ h^−1^ under one sun with reduced evaporation area. Moreover, the steam temperature can be as high as 95.5 °C under three sun intensities. This work discusses a progress for high‐temperature steam generation through a simple evaporation device. On the one hand, it can solve the demand for high‐quality water, on the other hand, it can expand the application of photothermal interfacial evaporation in sterilization and disinfection.

## 2 Results and Discussion

### 2.1 Fabrication Process and Characterization

**Figure 1:**
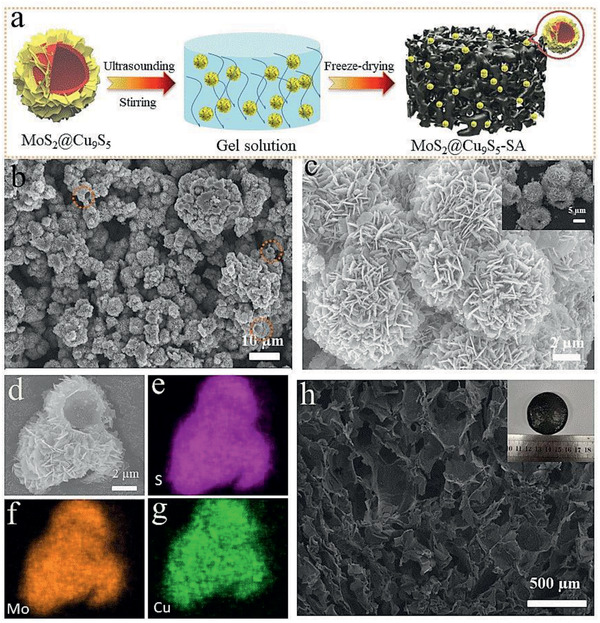
a) Simple illustration for describing the synthesis process of MoS_2_@Cu_9_S_5_‐SA. b,c) SEM image of MoS_2_@Cu_9_S_5_. d–g) Elemental mapping analysis of S, Cu, and Mo for MoS_2_@Cu_9_S_5_. h) The morphology image of MoS_2_@Cu_9_S_5_‐SA.

Based on previous reports, molybdenum sulfide‐based photothermal materials have made favorable progress in solar interfacial evaporation filed.^[^ ^33^, ^34^, ^35^, ^36^, ^37^, ^38^ ^]^ Compared with Ti_2_O_3_,^[^ ^39^ ^]^ gold,^[^ ^40^, ^41^, ^42^ ^]^ carbon‐based materials,^[^ ^13^, ^15^, ^18^, ^21^ ^]^ etc., molybdenum sulfide‐based photothermal materials have two advantages: i) In terms of preparation, the raw material sources are relatively wide. The preparation method is simple and the cost is relatively low; ii) The morphology of molybdenum sulfide‐based photothermal materials is generally curdled structure, which is conducive to improving internal refraction of light, reducing light reflection, and improving light absorption.^[^ ^43^ ^]^ Here, we developed MoS_2_@Cu_9_S_5_ composite as a photothermal material for enhanced solar steam generation. The photothermal material was prepared by the synthesis of MoS_2_@Cu_9_S_5_ and molding of 3D MoS_2_@Cu_9_S_5_‐SA hydrogel. First, we synthesized polyhedral Cu_2_O as a template and precursor (Figure S1, Supporting Information, for scanning electron microscopy, SEM image of Cu_2_O). MoS_2_@Cu_9_S_5_ was prepared through two steps of hydrothermal and annealing. After hydrothermal and annealing processes, Cu_2_O was sulfided into Cu_9_S_5_, and at the same time, sheet MoS_2_ was grown on it. The 3D MoS_2_@Cu_9_S_5_‐SA hydrogel was developed by solution stirring and freeze‐drying processes, which is shown in **Figure** **1**a. In Figure 1b,c, the SEM image of MoS_2_@Cu_9_S_5_ displays that the sheet‐like MoS_2_ is arranged on the polyhedral spherical structure to form a hollow flower ball structure. The structure is advantageous for enhanced light absorption by multiple internal refraction of light.^[^ ^43^ ^]^ The elemental mapping (Figure 1d–g) reveals that S, Mo, and Cu atoms present a uniform distribution on the MoS_2_@Cu_9_S_5_. In addition, the morphology of MoS_2_@Cu_9_S_5_‐SA was characterized and shown in Figure 1h. It reveals an interconnected porous network structure inside MoS_2_@Cu_9_S_5_‐SA, which is very beneficial for steam escape and water supply during the solar interfacial evaporation.

**Figure 2:**
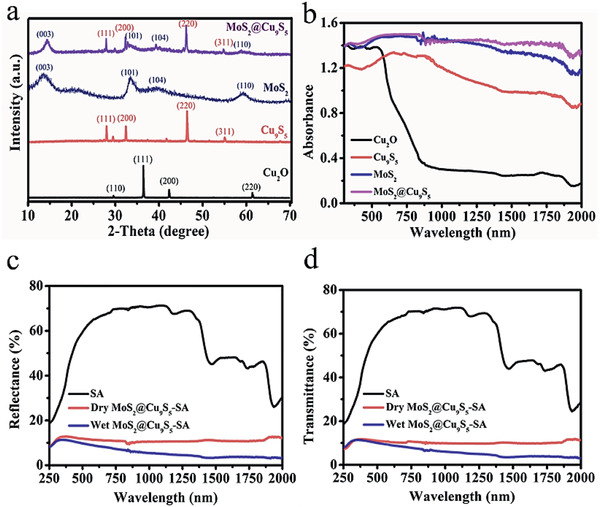
a) XRD patterns of Cu_2_O, Cu_9_S_5_, MoS_2_, and MoS_2_@Cu_9_S_5_. b) Ultraviolet‐visible‐near‐infrared absorption spectrum of Cu_2_O, Cu_9_S_5_, MoS_2_, and MoS_2_@Cu_9_S_5_ (powder absorption). c) Light reflectance data of SA, dry MoS_2_@Cu_9_S_5_‐SA, and wet MoS_2_@Cu_9_S_5_‐SA. d) Light transmittance of SA, dry MoS_2_@Cu_9_S_5_‐SA, and wet MoS_2_@Cu_9_S_5_‐SA.

In order to better highlight the photothermal advantage of MoS_2_@Cu_9_S_5_, Cu_9_S_5_, and MoS_2_ was introduced as comparative photothermal materials (the Supporting Information for detailed preparation process). Cu_9_S_5_ is a hollow spherical structure with irregular surface (Figure S2, Supporting Information) and MoS_2_ shows a flower globular structure (Figure S3, Supporting Information). In **Figure** **2**a, X‐ray diffraction (XRD) patterns reveal that the peaks of Cu_2_O appear at 29.6°, 36.4°, 42.3°, and 61.4°, which are assigned to the (110), (111), (200), and (220) planes of Cu_2_O (JCPDS No. 017‐2174). The peaks at 27.8°, 32.2°, 46.2°, and 54.8° correspond to the (111), (200), (220), and (311) planes of Cu_9_S_5_ (JCPDS No. 005‐7213). The MoS_2_ prepared reveals distinct peaks at 14.2°, 33.2°, 38.7°, and 58.5° corresponding to the (003), (101), (104), and (110) planes of MoS_2_ (JCPDS No. 007‐6370). For MoS_2_@Cu_9_S_5_, XRD patterns clearly reveal that the peaks of MoS_2_ at 14.2°, 33.2°, 38.7°, and 58.5° and peaks of Cu_9_S_5_ at 27.8°, 32.2°, 46.2°, and 54.8°. At the same time, some weak peaks correspond to the peaks of Cu_2_O, probably due to the incomplete sulfuration of Cu_2_O. Figure 2b shows the comparison of light absorption for Cu_2_O, Cu_9_S_5_, MoS_2_, and MoS_2_@Cu_9_S_5_ in the wavelength range of 300–2000 nm. Compared with other samples, the MoS_2_@Cu_9_S_5_ obviously shows stronger light absorption especially in the infrared region. On the basis, the light reflectance and transmittance of MoS_2_@Cu_9_S_5_‐SA was further characterized. As shown in Figure 2c,d, pure dry SA has strong light reflectance and transmittance, but dry MoS_2_@Cu_9_S_5_‐SA only shows less than 10% light reflectance and transmittance across the spectral range of 300–2000 nm. Further, the wet MoS_2_@Cu_9_S_5_‐SA shows weaker light reflectance and transmittance. Two reasons can be explained: water can deepen the MoS_2_@Cu_9_S_5_‐SA color, and in addition, water also has a certain amount of light absorption.

### 2.2 Evaluation of Solar Interfacial Evaporation for MoS_2_@Cu_9_S_5_‐SA

**Figure 3:**
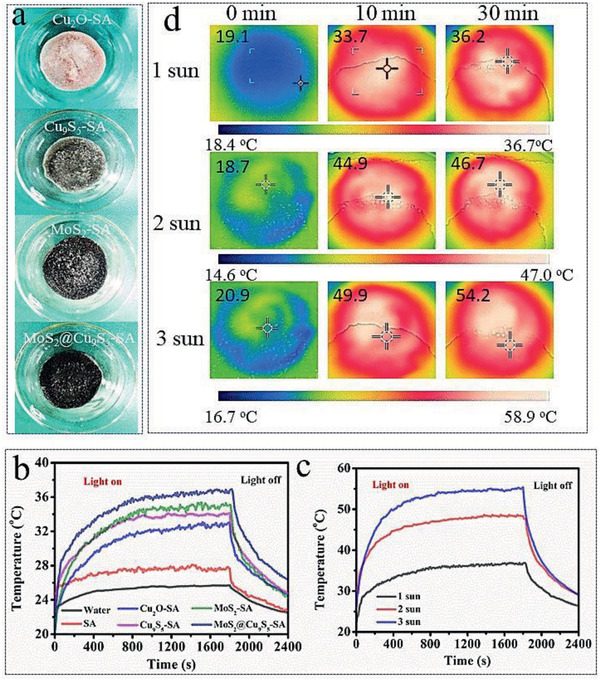
a) The photos of Cu_2_O‐SA, Cu_9_S_5_‐SA, MoS_2_‐SA, and MoS_2_@Cu_9_S_5_‐SA. b) Surface temperature change of water, SA, Cu_2_O‐SA, Cu_9_S_5_‐SA, MoS_2_‐SA, and MoS_2_@Cu_9_S_5_‐SA with time under one sun radiation. c) Surface temperature change of MoS_2_@Cu_9_S_5_‐SA with time under different light intensities. d) The infrared images of MoS_2_@Cu_9_S_5_‐SA under different light intensities.

Solar interfacial evaporation experiment was carried out to further verify good photothermal performance of MoS_2_@Cu_9_S_5_‐SA. First, we compared the photothermal properties of different amounts of MoS_2_@Cu_9_S_5_ (10, 20, 30, 40, 50, and 60 mg) incorporated into SA. As the quality of MoS_2_@Cu_9_S_5_ increases, the mass loss of evaporation gradually increases. As shown in Figure S4 in the Supporting Information, after half an hour, the evaporation weight changes reach 0.454 kg m^−2^ (10 mg), 0.552 kg m^−2^ (20 mg), 0.646 kg m^−2^ (30 mg), 0.690 kg m^−2^ (40 mg), 0.774 kg m^−2^ (50 mg), and 0.769 kg m^−2^ (60 mg). When the quality of MoS_2_@Cu_9_S_5_ is increased to 50 and 60 mg, the mass loss of the evaporated water is almost the same. It is meaningless to increase the amount of photothermal material, so 50 mg is selected as the optimal amount to add SA. Then the photothermal properties of different materials (water, SA, Cu_2_O‐SA, Cu_9_S_5_‐SA, MoS_2_‐SA, and MoS_2_@Cu_9_S_5_‐SA (**Figure** **3**a for the photos of Cu_2_O‐SA, Cu_9_S_5_‐SA, MoS_2_‐SA, and MoS_2_@Cu_9_S_5_‐SA)) were also tested. Figure 3b discloses the surface temperature changes of different photothermal materials with time of illumination under one sun. The temperature–time curve reveals that MoS_2_@Cu_9_S_5_‐SA has rapid photothermal response at the moment of the light on. The surface temperature of MoS_2_@Cu_9_S_5_‐SA eventually is stabilized at ≈36.5 °C. However, the surface temperatures of Cu_2_O‐SA, Cu_9_S_5_‐SA, and MoS_2_‐SA are ≈32.6, ≈34.0, and ≈35.1 °C, and only ≈25.6 and ≈27.5 °C for water and SA. The surface temperature tests prove that MoS_2_@Cu_9_S_5_‐SA has a stronger ability for transforming light to heat than Cu_2_O‐SA, Cu_9_S_5_‐SA, and MoS_2_‐SA. Further, the surface temperatures of MoS_2_@Cu_9_S_5_‐SA can reach ≈36.5, ≈48.3, and ≈55.1 °C under one, two, and three sun, respectively (Figure 3c). The infrared images (Figure 3d) further show that the surface temperature of MoS_2_@Cu_9_S_5_‐SA under one, two, and three sun, and the results are consistent with the results of thermocouple tests. The surface temperature tests prove the rapid light‐to‐heat response and good photothermal performance of MoS_2_@Cu_9_S_5_‐SA.

**Figure 4:**
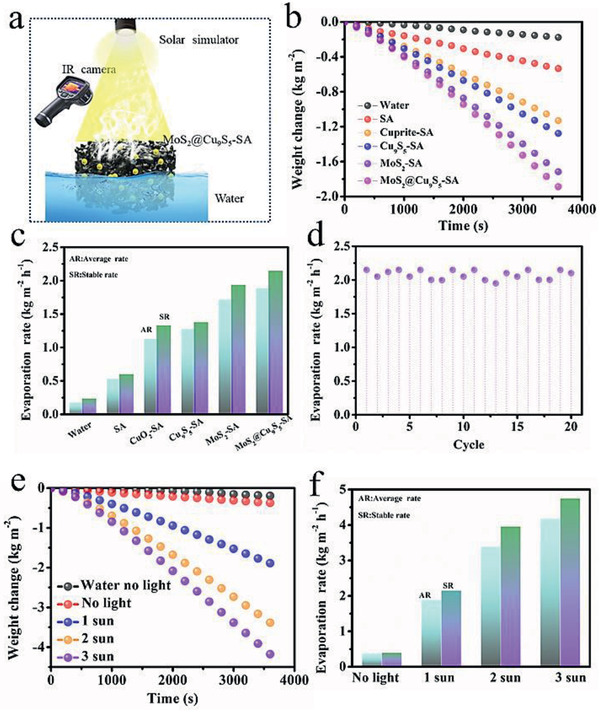
a) Simple illustration for solar interfacial evaporation. b) Real‐time weight changes of water, SA, Cu_2_O‐SA, Cu_9_S_5_‐SA, MoS_2_‐SA, and MoS_2_@Cu_9_S_5_‐SA under one sun. c) Evaporation rate of water, SA, Cu_2_O‐SA, Cu_9_S_5_‐SA, MoS_2_‐SA, and MoS_2_@Cu_9_S_5_‐SA after an hour of exposure to one sun. d) Cycle tests of MoS_2_@Cu_9_S_5_‐SA under one sun. e) The weight change of evaporation with MoS_2_@Cu_9_S_5_‐SA under different light densities. f) Evaporation rate with MoS_2_@Cu_9_S_5_‐SA under different light densities.

**Figure** **4**a shows a simple diagram of solar interfacial evaporation. The MoS_2_@Cu_9_S_5_‐SA floats on the surface of the water, generates local heat under the sunlight, and steam is quickly generated. The weight change–time curve reveals the water weight loss of solar interfacial evaporation. As shown in Figure 4b and Table S1 in the Supporting Information, after an hour of exposure to one sun, the weight changes of water, SA, Cu_2_O‐SA, Cu_9_S_5_‐SA, MoS_2_‐SA, and MoS_2_@Cu_9_S_5_‐SA are 0.176, 0.534, 1.131, 1.277, 1.716, and 1.888 kg m^−2^, respectively. Obviously, the MoS_2_@Cu_9_S_5_‐SA has the strongest solar interfacial evaporation capacity, which should be attributed to its strong light absorption and fast light response performance. After an hour, the evaporation rates of water, SA, Cu_2_O‐SA, Cu_9_S_5_‐SA, and MoS_2_‐SA are stabilized at 0.238, 0.603, 1.334, 1.380, and 1.940 kg m^−2^ h^−1^, respectively (Figure 4c). For MoS_2_@Cu_9_S_5_‐SA, the stable evaporation rate can reach 2.150 kg m^−2^ h^−1^, which is nine times higher than that of only water. Meanwhile, after cycle tests for 20 times, the photothermal evaporation rate still kept at ≈2.1 kg m^−2^ h^−1^ (Figure 4d). When the solar light intensity increases to two and three sun, after an hour, the weight changes of evaporation with MoS_2_@Cu_9_S_5_‐SA can reach 3.385 and 4.177 kg m^−2^ (Figure 4e). The evaporation rates can be stabilized at 3.959 kg m^−2^ h^−1^ for two sun and 4.748 kg m^−2^ h^−1^ for three sun (Figure 4f). In addition, in the absence of light, the MoS_2_@Cu_9_S_5_‐SA can also enhance evaporation and has a stronger evaporation performance than water, which may be caused by additional ambient heat.

### 2.3 Practical Application of Solar Interfacial Evaporation with MoS_2_@Cu_9_S_5_‐SA

**Figure 5:**
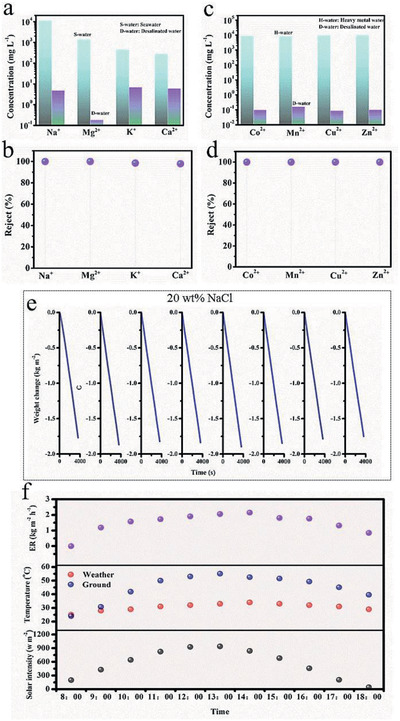
a) The ion concentration of Na^+^, K ^+^, Ca^2+^, and Mg^2+^ of seawater and desalinated water. b) The ion rejection of seawater. c)The ion concentration of Co^2+^, Mn^2+^, Cu^2+^, Zn^2+^ before and after purification. d) The ion rejection of sewage containing heavy metal ions. e) Long‐time photothermal test with high‐concentration brine. f) Solar interfacial evaporation under natural sun‐light from 8:00 to 18:00 (September 3, 2020).

The practical application of solar interfacial evaporation with MoS_2_@Cu_9_S_5_‐SA for water remediation is shown in **Figure** **5**. The ions concentration was detected by inductively coupled plasma optical emission spectrometer. After desalination, the concentration of Na^+^, Mg^2+^, K^+^, and Ca^2+^ in seawater decrease from 11 280, 1394, 453.5, and 279 mg L^−1^ to 4.79, 0.18, 6.86, and 5.96 mg L^−1^ (Figure 5a) with ions concentration below the drinking standard of the World Health Organization, and the ion rejection can achieve 99.95%, 99.98%, 98.5%, and 97.9% (Figure 5b), respectively, suggesting that the efficient desalination performance of MoS_2_@Cu_9_S_5_‐SA. In addition, heavy metal ions pollution is also one of the important factors of water pollution. After desalination, the ions concentration of sewage containing Co^2+^, Mn^2+^, Cu^2+^, and Zn^2+^ decrease from 8835, 8241, 9532.5, and 9807 mg L^−1^ to 0.098, 0.16, 0.087, and 0.1 mg L^−1^ (Figure 5c) with a high rejection of more than 99.9% (Figure 5d), suggesting the high‐efficiency purification ability of MoS_2_@Cu_9_S_5_‐SA for sewage to meet the standard of drinkable water. Meanwhile, the MoS_2_@Cu_9_S_5_‐SA also demonstrated the high‐efficiency photothermal interface evaporation stability in high‐concentration brine. As shown in Figure 5e, after photothermal test for 8 h in 20 wt% NaCl solution, the evaporation rate can still remain at 1.75 kg m^−2^ h^−1^. Finally, the photothermal interface evaporation test of MoS_2_@Cu_9_S_5_‐SA under natural solar light is shown in Figure 5f. The solar heat flux was 43–940 kW m^−2^ from 8:00 to 18:00 with ground temperature of 24–55.1 °C and temperature by weather forecast of 25–34 °C (September 3, 2020). The water interfacial evaporation rate can reach ≈2.1 kg m^−2^ h^−1^ at noon. The water produced on this day can reach 16.5 kg m^−2^, which is capable of meeting daily drinking water needs. In addition, after 1 year, the MoS_2_@Cu_9_S_5_‐SA still maintains low light reflection and transmission (Figure S5, Supporting Information) and the continuous tests in high‐concentration salt water (20 wt% NaCl solution) for up to 10 h can still maintain high evaporation rate up to ≈1.74 kg m^−2^ h^−1^ (Figure S6, Supporting Information), and there is almost no salt deposit on the surface of MoS_2_@Cu_9_S_5_‐SA.

### 2.4 Three‐Tier Device for Enhanced Steam Temperature

**Figure 6:**
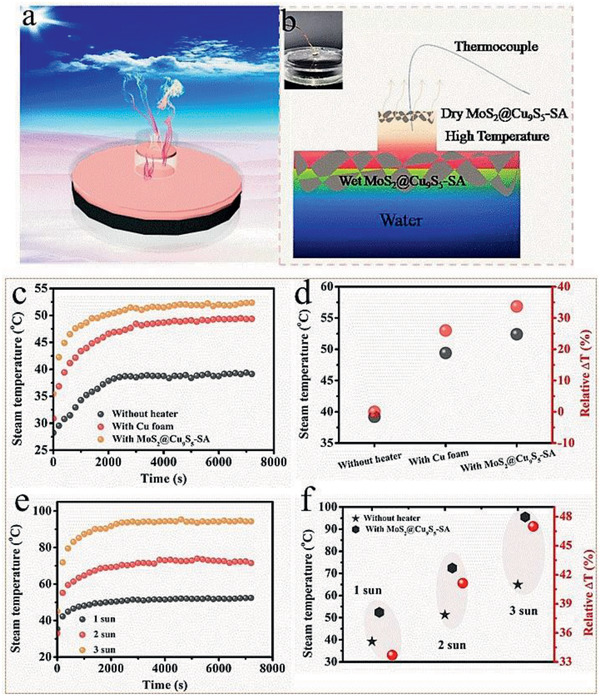
a) A simple diagram of the three‐tier device for enhanced steam temperature. b) A detailed description picture of the three‐tier device with enhanced steam temperature. c) The change of steam temperature without heater, with Cu foam as the upper heater and MoS_2_@Cu_9_S_5_‐SA as the upper heater under one sun. d) Percentage increase in steam temperature of with Cu foam as the upper heater and MoS_2_@Cu_9_S_5_‐SA as the upper heater than that of without heater under one sun. e) Steam temperature with MoS_2_@Cu_9_S_5_‐SA under different light intensities. f) Percentage increase in steam temperature with MoS_2_@Cu_9_S_5_‐SA than that of without heater under different light densities.

It is known from previous studies that dry photothermal materials will have a much higher temperature than wet ones under light.^[^ ^44^, ^45^ ^]^ Inspired by this, a three‐tier device was designed for solar steam generation with enhanced steam temperature. **Figure** **6**a shows a simplified diagram of the three‐tier device. The dry MoS_2_@Cu_9_S_5_‐SA is placed at the top of the steam outlet as a heater and the wet MoS_2_@Cu_9_S_5_‐SA is placed at in water as a photothermal evaporation layer. The air gap between the dry MoS_2_@Cu_9_S_5_‐SA and the wet MoS_2_@Cu_9_S_5_‐SA will become a high‐temperature area, and the steam can be heated again, thereby increasing the steam temperature. A thermocouple is placed in the high‐temperature air gap area to test the steam temperature (Figure 6b).

The change of steam temperature with time was shown in Figure 6c. We compared the steam temperature changes without heater, with Cu foam as the upper heater and MoS_2_@Cu_9_S_5_‐SA as the upper heater. As shown in Figure 6d, the steam temperature without heater is just stabilized to ≈39.1 °C under one sun for 2 h. The steam temperatures with Cu foam and with MoS_2_@Cu_9_S_5_‐SA can heat up quickly and stabilize to ≈49.4 and ≈52.4 °C, which improve by 26% and 33.7% than that of without heater, respectively (Figure 6d). The evaporation rate of three‐tier device is reduced due to reduced area for steam escaping, but it can still maintain 1.04 kg m^−2^ h^−1^ under one sun (Figure S7, Supporting Information). Then the steam temperature was further tested under two sun and three sun (Figure 6e). After 2 h, the steam temperatures without heater are stabilized at ≈51.3 (Figure S8, Supporting Information) and ≈64.9 °C (Figure S9, Supporting Information) under two and three sun, while the steam temperatures with MoS_2_@Cu_9_S_5_‐SA can reach ≈72.4 and ≈95.5 °C, respectively. The steam temperature is increased by 41.1% and 47% (Figure 6f). This simple three‐tier device discusses the possibility of increasing the steam temperature.

## 3 Conclusion

In summary, inspired by the higher temperature of dry photothermal materials under sun illumination, the work develops a simple three‐tier device for solar steam generation with enhanced steam temperature. Here, we choose MoS_2_@Cu_9_S_5_‐SA as the photothermal material, which can achieve an interfacial evaporation rate of up to 2.15 kg m^−2^ h^−1^ under one sun illumination. On the basis of achieving good light absorption and excellent photothermal performance, we further design the simple three‐layer device (dry absorber–air gap–wet absorber). The air gap forms a high‐temperature area for reheating the steam generated by evaporation in the interface. The steam temperature can improve 33.7%, 41.13%, 47% of the device without dry absorber under one sun, two sun, three sun illumination, respectively. Moreover, the steam temperature can be as high as 95.5 °C under three sun intensities. Our work provides the possibility to solve the demand for high‐quality water and expand the application of photothermal interfacial evaporation in sterilization and disinfection. Simultaneously, this work will also increase some ideas for the subsequent researches for the in‐depth study of ultrahigh‐temperature steam by a simple method in the future.

## 4 Experimental Section

##### Materials

Copper sulfate pentahydrate (CuSO_4_·5H_2_O), sodium hydroxide (NaOH), ethanol, sodium molybdate dehydrate (Na_2_MoO_4_·H_2_O), urea, thioacetamide (C_2_H_5_NS), calcium carbonate (CaCO_3_), and glacial acetic acid (CH_3_COOH) were bought from sinopharm chemical Reagent Co., Ltd. d‐(+)‐Glucose monohydrate was bought from Aladdin Industrial Corporation #1008 Qigang Rd, Nanqiao Town, Fengxian (201406) Shanghai, China. SA was obtained from shanghai Macklin Biochemical Co., Ltd.

##### Preparation of Cu_2_O

First, 1.25 g of CuSO_4_·5H_2_O was added to 50 mL deionized water and heated to 50 °C under a magnetic stirring of 300 rad min^−1^. After 2 min, 30 mL of NaOH (3 m) solution was quickly poured into the above solution. The magnetic stirring speed was adjusted to 850 rad min^−1^ and the temperature was up to 70 °C. Then, 0.3 g d‐(+)‐glucose monohydrate was added to above solution and was keep at 70 °C for 20 min. The sample was cooled to room temperature, centrifuged with water and ethanol several times (10000 rpm, 10 min), and finally dried at 60 °C for 12 h, and saved for subsequent use.

##### Preparation of MoS_2_@Cu_9_S_5_

MoS_2_@Cu_9_S_5_ was synthesized by one‐step hydrothermal method. The prepared 40 mg Cu_2_O was added to the mixed liquid of 25 mL of ethanol and 15 mL of deionized water. Under stirring, 100 mg of Na_2_MoO_4_·H_2_O, 0.3 g of urea, and 70 mg of C_2_H_5_NS were added in above solution, followed by stirring for 30 min. Then, the mixed solution was transferred to 50 mL autoclave, sealed, and heated at 160 °C for 24 h. After cooling to room temperature, the sample was centrifuged with ethanol, deionized water for several times (10 000 rpm, 10 min), and dried at 60 °C in a vacuum drying oven. Finally, the sample was annealed at 300 °C in Ar. The heating rate and soaking time was 5 °C min^−1^ and 2 h, respectively.

##### Preparation of MoS_2_@Cu_9_S_5_‐SA

50 mg MoS_2_@Cu_9_S_5_ was ultrasonically dispersed in deionized water (30 mL), under stirring, added 0.5 g CaCO_3_, and 0.5 g SA to above solution, and kept magnetic stirring for 2 h. After the solution became viscous, it was poured into a beaker and frozen in a refrigerator for 24 h, then dried in the freezing‐dryer for 48 h. The dried gel was soaked in 20 mL CH_3_COOH solution (The volume ratio of CH_3_COOH and deionized water is 1:20), and then placed in the refrigerator to freeze for 24 h, and dried in the freezing‐dryer for 48 h.

##### Characterization

The morphology of sample was observed by a field‐emission scanning electron microscopy (JSM7100F). Following with SEM, elemental mapping was performed. XRD data of these samples were performed by an X‐ray diffractometer (Bruker, Germany) with a Cu K‐alpha radiation source. Ultraviolet‐visible‐near‐infrared spectrophotometer (Shimadze UV–vis–NIR UV‐3600 double beam spectrophotometer) was used to record the optical performance of these samples. Baseline calibration was carried out using barium sulfate before measuring absorbance, reflectance and transmission.

##### Evaluation of Solar Steam Evaporation

All samples were placed in a glass container containing 50 mL water for photothermal testing. Evaporation tests of the samples were evaluated by a solar simulator (PLS‐FX300HU) with a solar filter (AM1.5). The weight loss of water was monitored by an electronic balance with an accuracy of 0.0001 g and real‐time was recorded by computer. A full‐spectrum optical power meter (CEL‐NP2000‐2, Beijing Education Au‐light Co., Ltd.) was used to identify the light intensity. Surface temperatures of different samples were measured by thermocouples (OMEGA, INSP# 33306, 5TC‐TT‐K‐30‐197) and the values could be read by a data acquisition device (KEYSIGHT, 34972A). Infrared camera (FLIR E4 Pro, America) was used to further measure the surface temperature of the sample at different times.

##### Three‐Tier Device for Enhanced Steam Temperature

The three‐tier device consists of wet absorber, air gap and dry absorber. Wet absorber in direct contact with water, the air gap is about 3 mm. The dry absorber is placed at the top of the steam outlet as a heater and the thickness is 2 mm. The diameter of the glass container is 6 cm, the upper end is sealed during the test, and the diameter of the steam outlet is 1 cm. The steam temperature was measured by thermocouples (OMEGA, INSP# 33306, 5TC‐TT‐K‐30‐197) and the data could be read by a data acquisition device (KEYSIGHT, 34972A). The percentage increase in steam temperature can be calculated by the following formula

(1) $$\begin{array}{c} R=\frac{T_{2}-T_{1}}{T_{1}} \end{array}$$

where *R* is relative Δ*T* (%) of steam temperature. *T*_2_ and *T*_1_ are the steam temperature of with three‐tier device and without heater, respectively.

## Conflict of Interest

The authors declare no conflict of interest.

## Supporting information

Supporting Information

## Data Availability

Data openly available in a public repository that issues datasets with DOIs.
