# Supplementary material for: Enhanced Steam Temperature Enabled by a Simple Three‐Tier Solar Evaporation Device
Source: Glob Chall. 2021 Feb 18;5(4):2000092. doi: 10.1002/gch2.202000092 (PMC8025398; doi:10.1002/gch2.202000092)
Supplement: Supplementary file 1 — Supporting Information [file GCH2-5-2000092-s001.pdf]

© 2021 Wiley-VCH GmbH

Global Challenges
Open Access

Supporting Information

for *Global Challenges,* DOI: 10.1002/gch2.202000092

Enhanced Steam Temperature Enabled by a Simple Three-Tier Solar Evaporation Device

*Zhenzhen Guo, You Xu, Fang Yu, Jiacheng Yin, and Xianbao Wang**

Supporting information

Enhanced Steam Temperature Enabled by a Simple Three-tier Solar Evaporation Device

Zhenzhen Guo, You Xu, Fang Yu, Jiacheng Yin, Xianbao Wang*

Hubei Collaborative Innovation Center for Advanced Organic Chemical Materials, Key Laboratory for the Green Preparation and Application of Functional Materials, Ministry of Education, Hubei Key Laboratory of Polymer Materials, School of Materials Science and Engineering, Hubei University, Wuhan 430062, China

* Corresponding author. Tel.: +86 2788661729; fax: +86 2788661729.

E-mail addresses: wxb@hubu.edu.cn (Xianbao Wang)

Preparation of Cu_9S_5: The prepared 40 mg Cu_2O was added to the mixed liquid of 25 ml of ethanol and 15 mL of deionized water. Under stirring, 0.3 g of urea, and 70 mg of C_2H_5NS were added in above solution, followed by stirring for 30 minutes. Then transferring the mixed solution to 50 mL autoclave, sealed, and heated at 160 ^{o}C for 24 h. After cooling to room temperature, the sample was centrifuged with ethanol, deionized water for several times (10000rpm, 10 min), and dried at 60 ^{o}C in a vacuum drying oven. Finally, the sample was annealed at 300 ^{o}C in Ar. The heating rate and soaking time was 5 ^{o}C min^{-1} and 2 h, respectively.

Preparation of MoS_2: 200 mg of $Na_2MoO_4{\cdot}H_2O$, 0.6 g of urea, and 140 mg of C_2H_5NS were added in the mixed liquid of 25 ml of ethanol and 15 mL of deionized water,

followed by stirring for 30 minutes. Then transferring the mixed solution to 50 mL autoclave, sealed, and heated at 160 ^{o}C for 24 h. After cooling to room temperature, the sample was centrifuged with ethanol, deionized water for several times (10000rpm, 10 min), and dried at 60 ^{o}C in a vacuum drying oven. Finally, the sample was annealed at 300 ^{o}C in Ar. The heating rate and soaking time was 5 ^{o}C min^{-1} and 2 h, respectively.

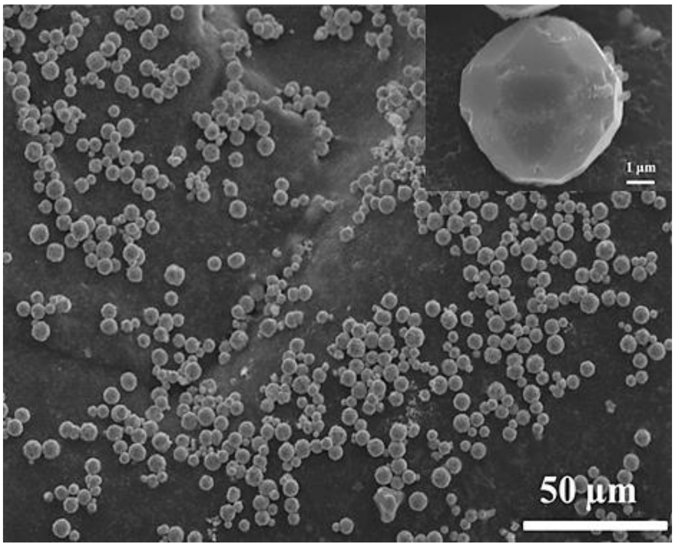

Figure S1. SEM image of Cu_2O

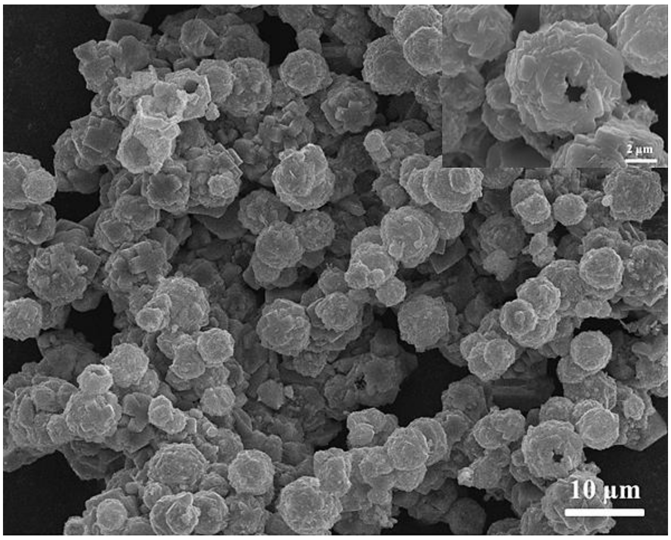

Figure S2. SEM image of Cu_9S_5.

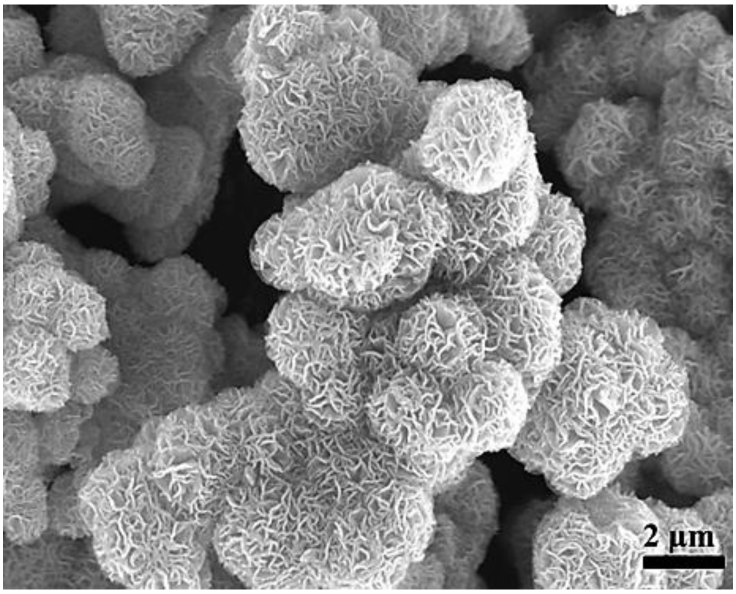

Figure S3. SEM image of MoS_2.

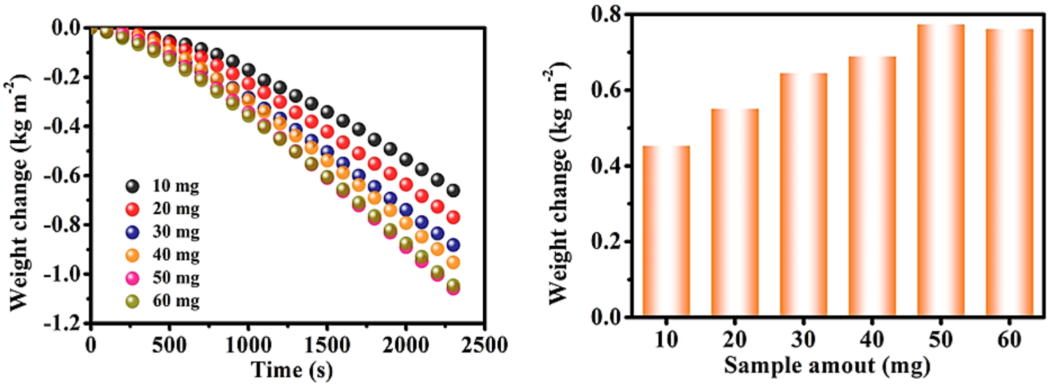

Figure S4. The weight change of water evaporated with different amount $MoS_2@Cu_9S_5$ (10 mg, 20 mg, 30 mg, 40 mg, 50 mg and 60 mg) incorporated into SA.

Table S1. The photothermal interfacial evaporation performance of different samples. The comparative data experiment was completed on the same day and under the same conditions. These samples were tested for one hour respectively.

Materials	Weight change (kg m^{-2})	Average evaporation rate (kg m^{-2} h^{-1})	Stable evaporation rate (kg m^{-2} h^{-1})
water	0.176	0.176	0.238
SA	0.534	0.534	0.603
CuO_2-SA	1.131	1.131	1.334
Cu_9S_5-SA	1.277	1.277	1.38
MoS_2-SA	1.716	1.716	1.94
MoS_2@Cu_9S_5-SA	1.888	1.888	2.15

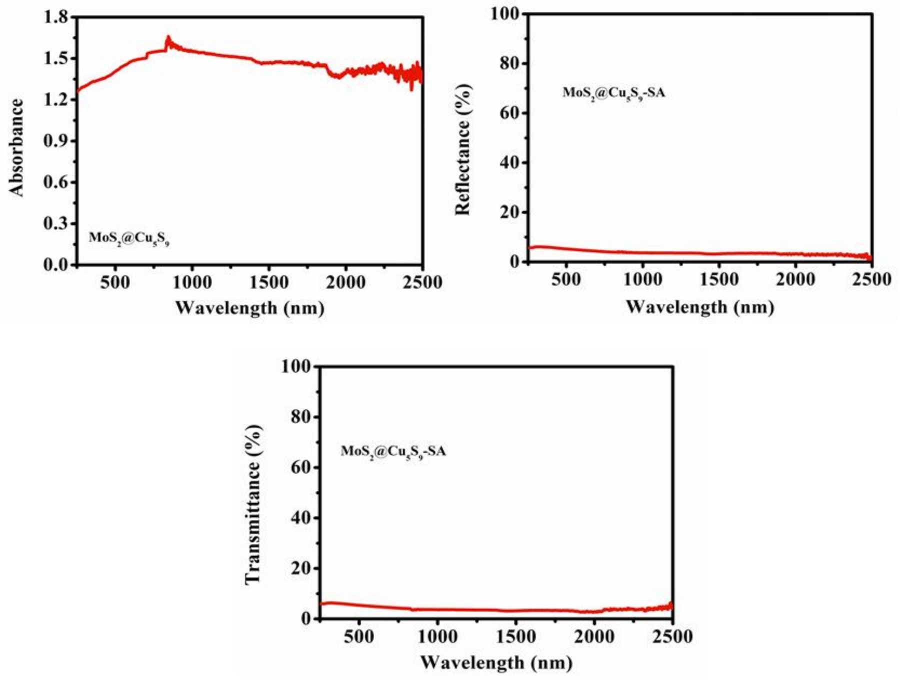

Figure S5. The light absorbance of MoS_2@Cu_9S_5 after one year. The light reflectance and transmittance of MoS_2@Cu_9S_5-SA after one year.

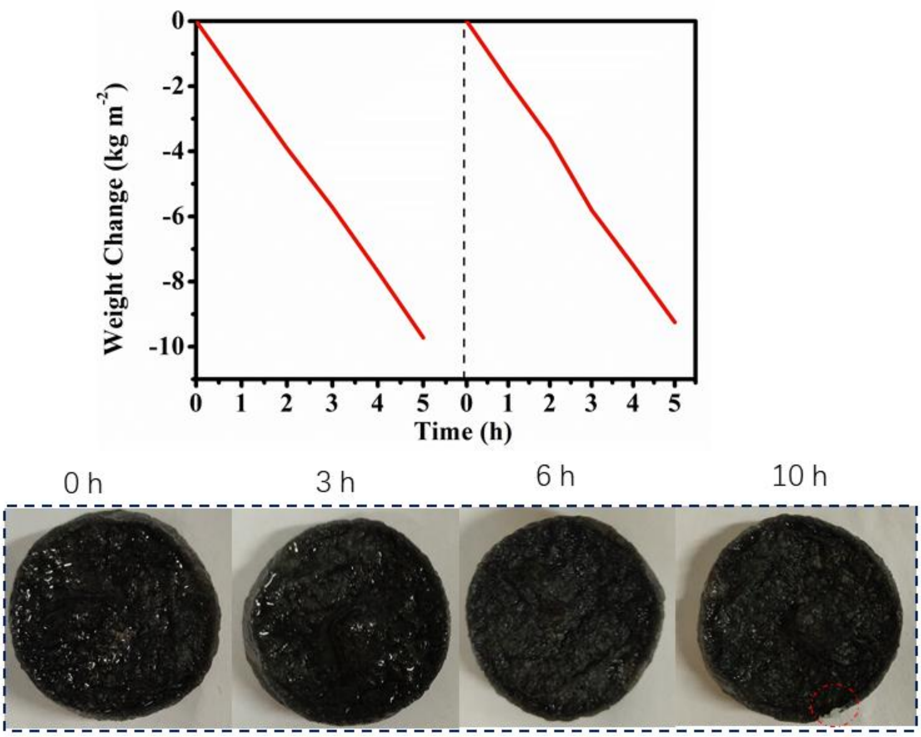

Figure S6. The photothermal performance of $MoS_2@Cu_9S_5$-SA after one year.

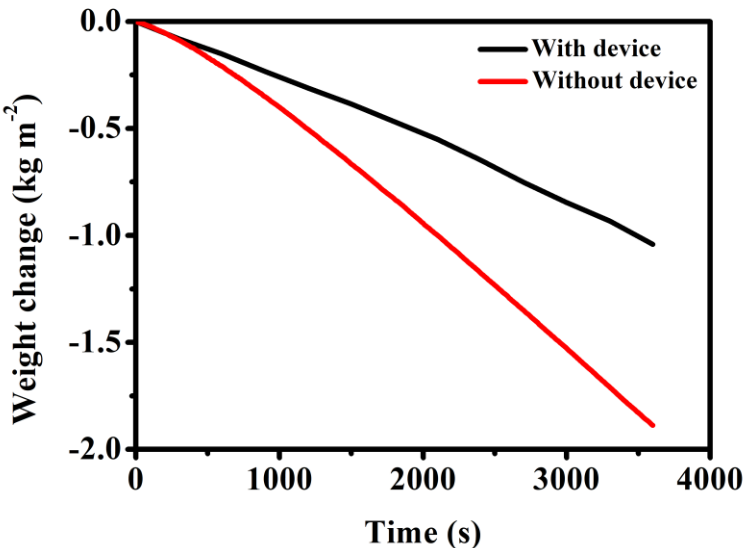

Figure S7. The weight change of evaporation water with and without three-tier device.

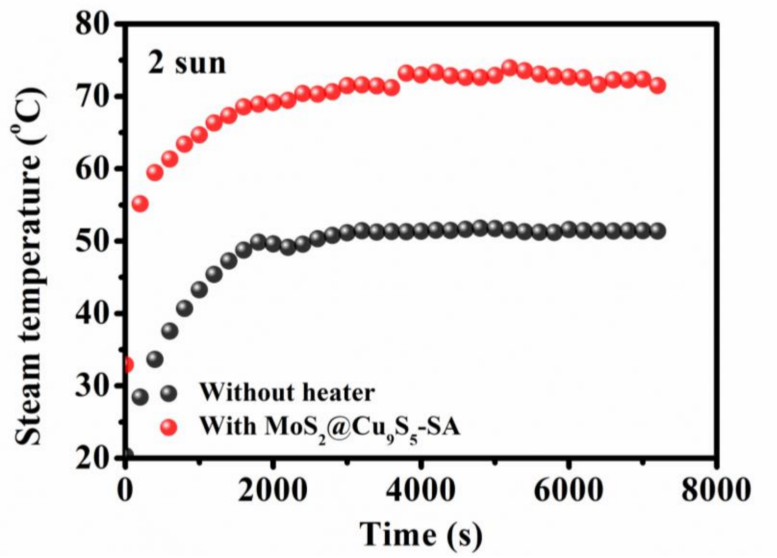

Figure S8. The change of steam temperature without heater and with $MoS_2@Cu_9S_5$-SA as the upper heater under two sun.

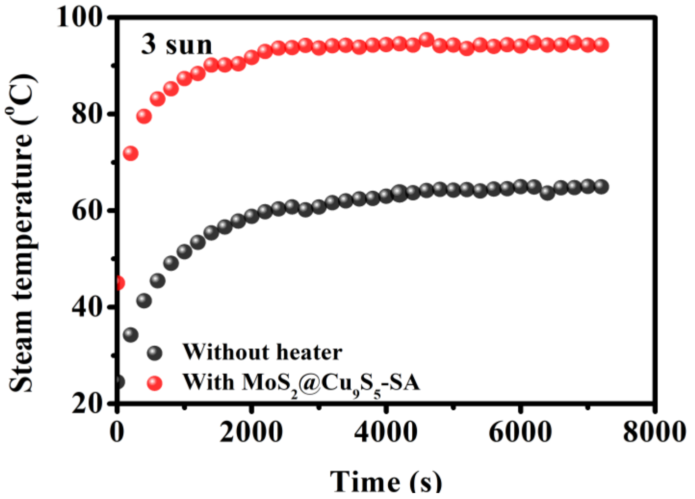

Figure S9. The change of steam temperature without heater and with $MoS_2@Cu_9S_5$-SA as the upper heater under three sun.
